# Kapβ2 Reverses Sevoflurane‐Induced Hydrogel Phase Transition of hnRNPA2/B1‐SG in Hypoxic Primary Rat Hippocampal Neurons

**DOI:** 10.1111/cns.70532

**Published:** 2025-08-07

**Authors:** Miao Zhang, Xinyi Wang, Feiyu Jia, Chenyi Yang, Zixuan Wang, Huihui Liao, Lin Zhang, Xi Xin, Haiyun Wang

**Affiliations:** ^1^ The Third Central Clinical College of Tianjin Medical University Tianjin China; ^2^ Nankai University Affinity the Third Central Hospital Tianjin China; ^3^ Tianjin Key Laboratory of Extracorporeal Life Support for Critical Diseases, Artificial Cell Engineering Technology Research Center Tianjin Institute of Hepatobiliary Disease Tianjin China

**Keywords:** hnRNPA2/B1, hydrogel phase transition, hypoxic hippocampal neurons, Kapβ2, neurodegenerative disorders, sevoflurane, stress granules

## Abstract

**Aims:**

Sevoflurane can aggravate the progression of neurodegeneration, although the underlying mechanisms remain incompletely understood. Our previous study identified a link between heterogeneous nuclear ribonucleoprotein A2/B1 (hnRNPA2/B1) and sevoflurane‐induced neurocognitive impairments. The abnormal hydrogel phase transition of stress granules (SGs) assembled via liquid–liquid phase separation (LLPS) by hnRNPA2/B1 is a crucial element in neurodegeneration. Karyopherin‐β2 (Kapβ2) is known to specifically recognize hnRNPA2/B1 and reverses the hydrogel transition of SGs. This study aimed to elucidate the mechanistic role of hnRNPA2/B1–SG phase transition in sevoflurane‐induced hippocampal neuronal dysfunction under hypoxic conditions, and to determine whether Kapβ2 can mitigate these effects.

**Methods:**

Using a hypoxic primary rat hippocampal neuron model and Kapβ2 overexpression, we investigated the effects of sevoflurane on hnRNPA2/B1 expression and subcellular distribution, phase separation dynamics, and the liquid‐to‐solid transition of hnRNPA2/B1‐associated SGs. We also assessed neuronal function and cognitive protein expression. Experimental approaches included Western blotting, RT‐qPCR, immunofluorescence staining, and fluorescence recovery after photobleaching (FRAP).

**Results:**

In hypoxic hippocampal neurons, sevoflurane altered the nuclear‐to‐cytoplasmic distribution of hnRNPA2/B1, promoted abnormal LLPS, and facilitated the formation of irreversible solid‐phase hnRNPA2/B1‐containing SGs. These changes were associated with neuronal dysfunction and reduced expression of cognition‐related proteins. Kapβ2 overexpression disrupted these aggregates, restored the dynamic reversibility of hnRNPA2/B1 LLPS, reversed the sevoflurane‐induced hydrogel phase transition of hnRNPA2/B1‐SGs, and enhanced the expression of cognition‐related proteins.

**Conclusion:**

The hydrogel phase transition of hnRNPA2/B1‐SG is a key pathological mechanism of sevoflurane‐induced hippocampal neuronal injury. Kapβ2 may serve as a potential therapeutic target to counteract sevoflurane‐related neurotoxicity.

## Graphical abstract

Sevoflurane exacerbates hippocampal neuronal dysfunction under hypoxic conditions by inducing the cytoplasmic mislocalization of hnRNPA2/B1, which drives abnormal liquid–liquid phase separation (LLPS) and subsequent irreversible hydrogel transition of hnRNPA2/B1‐containing stress granules (SGs). Kapβ2 reverses these pathological effects by dissolving aggregated hnRNPA2/B1, restoring its nuclear localization, and reestablishing the dynamic reversibility of SGs.

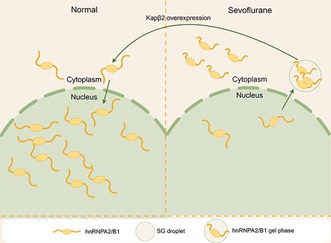

**Abbreviations**
AD: Alzheimer's disease
BDNF: brain‐derived neurotrophic factor
BSA: bovine serum albumin
CaMKII: calcium/calmodulin‐dependent protein kinase II
DIV: days in vitro
DMEM: Dulbecco's modified Eagle's medium
FRAP: Fluorescence recovery after photobleaching
GABAAR‐α1: Gamma aminobutyric acid A recept α1
GS: Goat serum
hnRNPA2/B1: heterogeneous nuclear ribonucleoprotein A2/B1
IDR: intrinsically disordered region
Kapβ2: Karyopherin‐β2
LLPS: liquid–liquid phase separation
MAC: minimum alveolar concentration
MOI: Multiply of Infection
NES: nuclear export signal
NIR: nuclear‐import receptors
NPC: nuclear pore complex
PBST: phosphate‐buffered saline 0.1% Tween‐20
PFA: paraformaldehyde
PrLDs: prion‐like domains
PS: penicillin–streptomycin
PY‐NLS: proline–tyrosine nuclear‐localization signals
RBP: RNA‐binding protein
RNP: ribonucleoprotein
RT‐qPCR: real‐time quantitative polymerase chain reaction
SG: stress granule
SIRT1: sirtuin 1
SPF: Specific Pathogen Free
TIA‐1: T‐cell intracellular antigen‐1

## 1 Introduction

Cerebrovascular pathology is commonly observed in the spectrum of neurodegenerative disorders [1]. The hippocampus, a critical brain region involved in cognitive processing, plays a central role in the progression of neurocognitive dysfunction [2]. Chronic cerebral brain hypoperfusion, which leads to hippocampal hypoxia, is considered a major pathological contributor to neurodegeneration [3, 4]. As the global population ages, an increasing number of elderly patients with chronic cerebral ischemia–hypoxia require anesthesia and surgical interventions to prevent disease. Sevoflurane, one of the most commonly used general anesthetics in elderly surgical patients, has been reported to exacerbate neurodegenerative pathology [5]. The molecular mechanisms underlying this effect may vary depending on the developmental stage of neurons and existing pathological conditions [6]. Thus, it is important to explore the impact of sevoflurane on cognition‐related hippocampal neurons under hypoxic conditions.

In neurodegenerative diseases such as Alzheimer's disease (AD) and frontotemporal dementia, a pathological phenomenon involves RNA‐binding proteins (RBPs) forming toxic, insoluble aggregates within stress granules (SGs) [7, 8]. Our previous study demonstrated that sevoflurane‐induced neurocognitive impairment is associated with nucleoplasmic translocation and cytoplasmic accumulation of heterogeneous nuclear ribonucleoprotein A2/B1 (hnRNPA2/B1) in the rat hippocampus [9]. HnRNPA2/B1 is a self‐nucleating, aggregation‐prone RBP [10] capable of forming reversible aggregates through self‐organization [11]. It contains intrinsically disordered regions (IDRs), low‐complexity domains (LCDs), and prion‐like domains (PrLDs), which facilitate liquid–liquid phase separation (LLPS) via multivalent weak interactions among proteins and RNAs, leading to the formation of liquid‐phase droplets [12]. LLPS is a phenomenon in which a supersaturated protein‐RNA mixture separates into coexisting dense and dilute phases [13]. In this context, the dense phase forms via self‐assembly of RBPs through their IDRs, and transient intermolecular interactions further recruit additional RNA and RBPs, expanding the dilute phase [14]. In vitro experiments exploring hnRNPA1 LLPS suggest that phase‐separated droplets can mature over time, undergoing a liquid–solid transition [15, 16] that compromises their dynamic fluidity [17]. This process has been implicated in the pathology of numerous neurodegenerative diseases [18].

HnRNPA2/B1 translocates from the nucleus to the cytoplasm [19] and initiates SGs assembly through LLPS under stress [20]. SGs, a prominent type of ribonucleoprotein (RNP) granule [10], are membrane‐free organelles composed of RNA and RBPs, formed in response to cellular stress [21]. HnRNPA2B1 demixes SGs into two stably co‐existing phases through LLPS: a dense phase, enriched for RBPs and RNA through IDRs self‐assembly, and a dilute phase. Super‐resolution microscopy has shown that SGs comprise a protein‐rich core surrounded by a dynamic shell of molecules that rapidly exchange with the surrounding cytosol [22]. The dense core acts as a selective compartment, regulating access of proteins and RNAs from the surrounding shell (dilute phase) [23]. The physical properties of SGs are strongly regulated by RBP and RNA abundance [24]. Abnormal LLPS impairs SG fluidity and drives pathological phase transitions [25, 26]. Under prolonged stress, SGs inhibit housekeeping gene translation and sequester mRNAs for storage, degradation, or re‐initiation until stress resolves [27]. However, persistent stress impairs LLPS dynamics, triggering an irreversible transition from a liquid to hydrogel states, facilitated by cross‐β junctions among aggregated molecules [28]. The dynamic nature of RNP granules, including SGs, is essential for their physiological function [29]. Dysregulation of this dynamic behavior and inappropriate gel‐like transitions have been implicated as key mechanisms accelerating neurodegenerative progression [29, 30]. Given this, we investigated whether sevoflurane affects the LLPS behavior and phase transitions of hnRNPA2/B1‐SGs in hypoxic hippocampal neurons.

The nuclear transport receptor Karyopherin‐β2 (Kapβ2), a member of the nuclear import receptor (NIR) family, specifically recognizes proline‐tyrosine nuclear‐localization signals (PY‐NLS) on RBPs. Kapβ2 disrupts cross‐β junctions, facilitating the nuclear import of RBPs through the nuclear pore complex (NPC) [31]. Additionally, Kapβ2 recognizes RNA recognition motifs (RRMs), arginine‐glycine–glycine‐rich (RGG) domains, and PrLDs on RBPs via low‐affinity interactions, enabling it to break weak non‐covalent bonds and efficiently reverse abnormal hydrogel states into liquid phases [32]. Studies have demonstrated that Kapβ2 can suppress cytoplasmic aggregation of FUS by preventing aberrant phase transitions in a PY‐NLS‐dependent manner [18]. Based on these findings, we sought to determine whether Kapβ2 could mitigate the adverse effects of sevoflurane by reversing the pathological phase transition of hnRNPA2/B1‐containing SGs.

## 2 Materials and Methods

### 2.1 Primary Hippocampal Neuron Culture and Hypoxia

Primary rat hippocampal neurons were isolated and cultured as previously described [33]. Hippocampi were dissected from postnatal day 0–1 rat pups and collected in high‐glucose Dulbecco's Modified Eagle's Medium (DMEM) supplemented with 1% penicillin–streptomycin (PS). The tissue was digested with 0.125% trypsin (Gibco) at 37°C for 15 min. Digestion was halted by adding planting medium (high‐glucose DMEM supplemented with 10% fetal bovine serum and 1% PS). The suspension was centrifuged at 1000 rpm for 5 min, the supernatant discarded, and the pellet resuspended in planting medium to generate a single‐cell suspension. Neurons were seeded at a density of 1 × 10^5^/mL into both confocal culture dishes and 6‐well plates. After cell adhesion (approximately 4 h post‐plating), the medium was replaced with growth medium (Neurobasal medium supplemented with 2% B27 and 1% GlutaMAX). On day 3 in vitro (DIV3), half of the growth medium was replaced, and cytarabine was added at a final concentration of 20 μM to inhibit glial proliferation. On DIV7, the medium was fully replaced with fresh growth medium, and thereafter, half of the culture medium was refreshed every 3 days.

Neurons were cultured until DIV21, then subjected to a hypoxia custom‐made closed cuboid culture chamber placed in a 37°C sterile incubator. Hypoxia was induced using an Ohmeda Excel 210 anesthesia machine. The N_2_O outlet was connected to CO_2_, the O_2_ outlet to N_2_, and the Air outlet to Air. The hypoxic gas mixture (3% O_2_, 92% N_2_, and 5% CO_2_) [34] was delivered through a gas filter (artificial nose) into the chamber. The outlet was connected to a gas monitor (Datex‐Ohmeda, Germany), maintaining oxygen levels at 3%–4%. Neurons were exposed to hypoxia for 3 h.

### 2.2 Drug Administration and Kapβ2 Overexpression in Hypoxic Neurons

Hypoxia neurons were randomly assigned to two groups. One group received no further treatment (control group; Hypoxia.Neu‐ctrl), while the other was exposed to 1.3 minimum alveolar concentration (MAC) sevoflurane for 3 h in the same hypoxic chamber (sevoflurane group; Hypoxia.Neu‐sev). The volatile tank of the anesthesia machine was accurately adjusted. Sevoflurane was introduced via the N_2_O vent of the anesthesia machine, passed through the artificial nose, and delivered to the chamber. The outlet was connected to an anesthesia monitor to maintain the sevoflurane concentration at 1.3 MAC.

**FIGURE 1:**
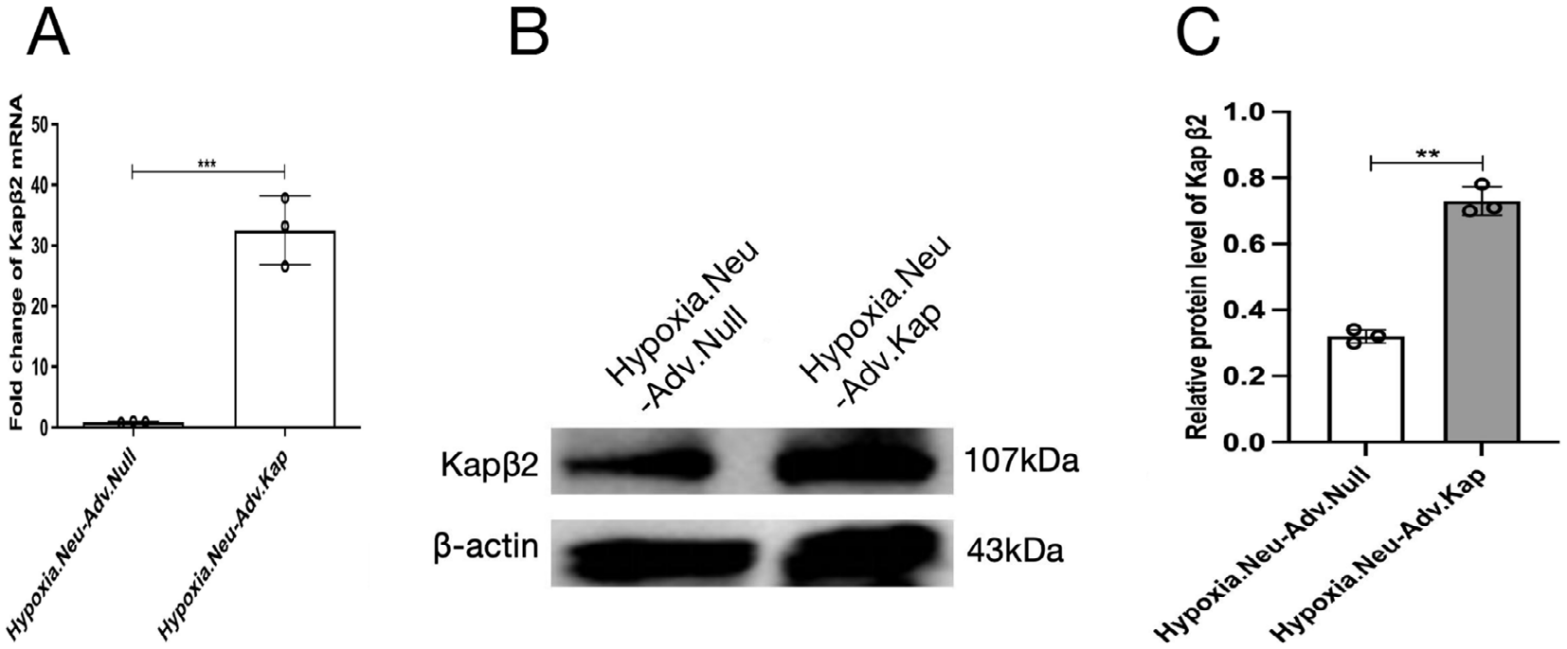
(A) The mRNA level of Kapβ2 in the hypoxic hippocampal neurons was detected by RT‐qPCR after 48 h of adenovirus infection. (B, C) The protein level and statistical analysis of Kapβ2 in the hypoxic hippocampal neurons were detected by Western blotting after 48 h of adenovirus infection. Data are presented as mean ± SD (*n* = 3/group), Student's *t*‐test was used to analyze results of qPCR and blots. **p < 0.01, ***p < 0.001 were considered significant.

Recombinant adenoviral vectors encoding Kapβ2 (Adv.Kapβ2) and control vectors (Adv.Null) were obtained from GenePharma (Shanghai, China). On DIV18, neurons were infected with either Adv.Kapβ2 or Adv. Null (Multiply of Infection [MOI] = 50) for 4 h. Afterward, the viral medium was removed and replaced with fresh growth medium for 48 h. Expression of Kapβ2 mRNA and protein was confirmed by RT‐qPCR and Western blotting (Figure 1A–C). On DIV21, adenovirus‐infected neurons were exposed to hypoxia for 3 h and then divided into four groups for sevoflurane treatment and subsequent analysis: (1) empty adenovirus, no sevoflurane (Hypoxia.Neu‐Adv.Null‐ctrl); (2) Kapβ2 adenovirus, no sevoflurane (Hypoxia.Neu‐Adv.Kap‐ctrl); (3) empty adenovirus, +1.3 MAC sevoflurane (Hypoxia.Neu‐Adv.Null‐sev); (4) Kapβ2 adenovirus, +1.3 MAC sevoflurane (Hypoxia.Neu‐Adv.Kap‐sev).

### 2.3 Plasmid Transfection and Fluorescence Recovery After Photobleaching

On DIV20, adenovirus‐treated neurons were transfected with hnRNPA2B1‐mCherry and/or TIA1‐EGFP plasmids (Hanheng Biotechnology Co. Ltd., Shanghai, China) using Lipofectamine 2000 (Invitrogen, USA) at a 2:1 reagent‐to‐plasmid (μL/μg) ratio. Briefly, 4 μL of Lipofectamine 2000 was mixed with 250 μL Neurobasal medium and incubated for 5 min at room temperature. In parallel, 6 μL of hnRNPA2B1‐mCherry (500 ng/μL) or 3 μL each of hnRNPA2B1‐mCherry and TIA1‐EGFP were diluted in 250 μL Neurobasal medium and also incubated for 5 min. The two solutions were combined and incubated for 20 min at room temperature before being added to 1.5 mL of culture medium in confocal dishes. After 6 h, the medium was replaced with fresh growth medium. At 24 h post‐transfection, neurons were subjected to hypoxia and then divided into corresponding drug treatment groups.

Fluorescence recovery after photobleaching (FRAP), which measures the diffusion of protein molecules within individual LLPS droplets, was performed using a confocal microscope (LSM 900; Carl Zeiss, Oberkochen, Germany) with a 63× oil‐immersion objective (NA 1.4, 1024 × 1024 resolution, 3.0× zoom, gain = 750). The fluorescence from mCherry was excited with a 561 nm laser; EGFP (TIA1) with a 488 nm laser. FRAP protocol: 5 s pre‐bleach, 15 ms bleach, and 25 s post‐bleach at 2 frames/s. The first image captured prior to photobleaching was used to calculate the initial fluorescence intensity, followed by 20 consecutive images in which bleaching and recovery were recorded. Bleaching was performed in a circular regions of interest (ROIs) (pinhole diameter at 4 Airy units) located at the center of the field of view using a 516 nm laser at 30% power. FRAP data were corrected for background. Fluorescence intensity in each ROI was measured after subtraction of background fluorescence, and the average in each time point within bleached ROIs was normalized to pre‐bleached intensity. FRAP curves were generated from the average of 13 cells, with 1–2 bleached regions per cell.

### 2.4 Immunofluorescence Staining

Hypoxic neurons from different groups were washed with phosphate‐buffered saline with 0.1% Tween‐20 (PBST), permeabilized in 1% Triton X‐100 in PBS for 30 min, and incubated with 10% goat serum (GS) or 5% bovine serum albumin (BSA) for 30 min at 37°C. Samples were incubated overnight at 4°C with primary antibodies: rabbit anti‐hnRNPA2B1 (1:4000; Abcam, ab259894, Cambridge, UK) and anti‐TIA1 antibody (1:200; Santa Cruz, sc‐166,247, Houston, TX, USA). After washing, secondary antibodies were applied for 2 h at 37°C: Alexa Fluor 594 goat anti‐rabbit (1:200; Abcam) and Alexa Fluor 488‐conjugated mouse κ IgG (1:50; Santa Cruz Biotechnology, sc‐516,176). Samples were stained with DAPI (8 min) and mounted using anti‐fluorescence quenching sealing solution (Beyotime, Haimen, China). Immunofluorescence staining was performed using a 100× oil‐immersion objective lens of a confocal microscope (Leica STELLARIS1.8).

### 2.5 Western Blotting

Neurons (1–2 million cells) were harvested in PBS, lysed in RIPA buffer with protease/phosphatase inhibitors, and incubated on ice for 30 min. Lysates were centrifuged at 16,000 ***g*** for 10 min at 4°C. Nuclear and cytoplasmic proteins were extracted (KGP150; KeyGEN; China), according to the manufacturer's instructions. The supernatants were denatured by boiling for 15 min at 100°C. Protein concentrations were quantified using a BCA Assay Kit (Beyotime; China). Equal amounts of protein (10–30 μg) per sample were loaded onto 4%–20% Mini‐PROTEAN TGX Precast Protein Gels and run in an SDS running buffer at 150 V for 1 h. Primary antibodies were as follows: β‐actin (1:1000, Affinity Biosciences, AF7018, China), Histone H3 (1:1000, Affinity Biosciences, AF0863, China), Kapβ2 (1:1000, Affinity Biosciences, DF13291, China), GABAAα1 (1:5000, Abcam, ab33299, UK), CAMK‐II (1:3000, Abcam, ab52476, UK), hnRNPA2B1 (1:9000, Abcam, ab183654, UK). Histone H3 was used for detection to control for equal nuclear protein loading, and β‐actin was used as the loading control for other blots. Membranes were visualized using an enhanced chemiluminescence detection kit (Millipore, Billerica, MA, USA). The bands were scanned and quantified using ImageJ.

### 2.6 Real‐Time Quantitative Polymerase Chain Reaction (RT‐qPCR)

Total RNA was extracted using TRIzol reagent (Invitrogen, USA). The mRNA levels of the target genes were measured through real‐time quantitative PCR (RT‐qPCR). Primers targeting β‐actin, GABAARα1, and CAMK‐II were acquired from Sangong Biotech (Shanghai, China). The sequences were as follows: β‐actin, 5′‐GCTGTGCTATGTTGCCCTAGACTTC‐3′ (sense) and 5′‐GGAACCGCTCATTGCCGATAGTG‐3′ (antisense); Kapβ2, 5′‐GGGATTGAAGAGGATGACGA‐3′ (sense) and 5′‐ACCCACTCGTGATGGAAAAG‐3′ (antisense); GABAARα1, 5′‐ACTGCTGGACGGTTATGACAATCG‐3′ (sense) and 5′‐GGTCTGAAACTGGTCCGAAACTGG‐3′ (antisense); CAMK‐II, 5′‐AGTCTCGTAAGCCCAGCCCAAG‐3′ (sense) and 5′‐ATCTTCGTCTTCTGTGGTGGTGTTG‐3′ (antisense). One microgram of RNA was reverse‐transcribed to first‐strand cDNAs using PrimeScript RT Master Mix (Takara, Japan). After denaturation, PCR amplification was conducted using the StepOne Real‐time PCR system with iTaq Universal SYBR Green Super Mix (Takara, Japan), as follows: 95°C for 30 s and up to 40 cycles of 95°C for 5 s and 60°C for 30 s in a 25 μL reaction system. The melting curve steps were 95°C for 15 s, 60°C for 15 s, and 95°C for 15 s. The β‐actin mRNA level was used as an internal control. All reactions were performed in triplicate. The relative mRNA levels were calculated using the 2^(− ΔΔCt)^ method.

### 2.7 Statistical Analysis

Data are expressed as mean ± standard deviation. Analyses were performed using SPSS 19.0 (version 19.0). Immunofluorescence images were quantified with ImageJ. Normality was assessed with the Shapiro–Wilk test, and variance homogeneity with Levene's test. For data meeting both assumptions (*p* > 0.05), parametric tests were appropriately used. Two‐group comparisons were conducted using independent‐sample *t*‐tests. For three or more groups, one‐way analysis of variance (ANOVA) was followed by the least significant difference (LSD) test for pairwise comparisons. Statistical significance was defined as *p* < 0.05. Sample size (*n* = 3) was determined based on: historical variability (CV < 5% from pilot studies), minimum detectable effect size (*d* > 1.5 at *α* = 0.05, *β* = 0.2), and alignment with cell biology standards [35].

## 3 Results

### 3.1 Sevoflurane Induces Ectopic Cytoplasmic Distribution of hnRNPA2/B1 in Hypoxia Hippocampal Neurons

Under normal physiological conditions, hnRNPA2/B1 is translocated from the nucleus to the cytoplasm, maintaining an equilibrium between the nucleus and cytoplasm [36]. However, in response to various internal and external stimuli, its nuclear re‐entry is often hindered [37]. To investigate the effects of sevoflurane on hnRNPA2/B1 distribution under hypoxic conditions, we performed Western blotting and immunofluorescence analysis.

**FIGURE 2:**
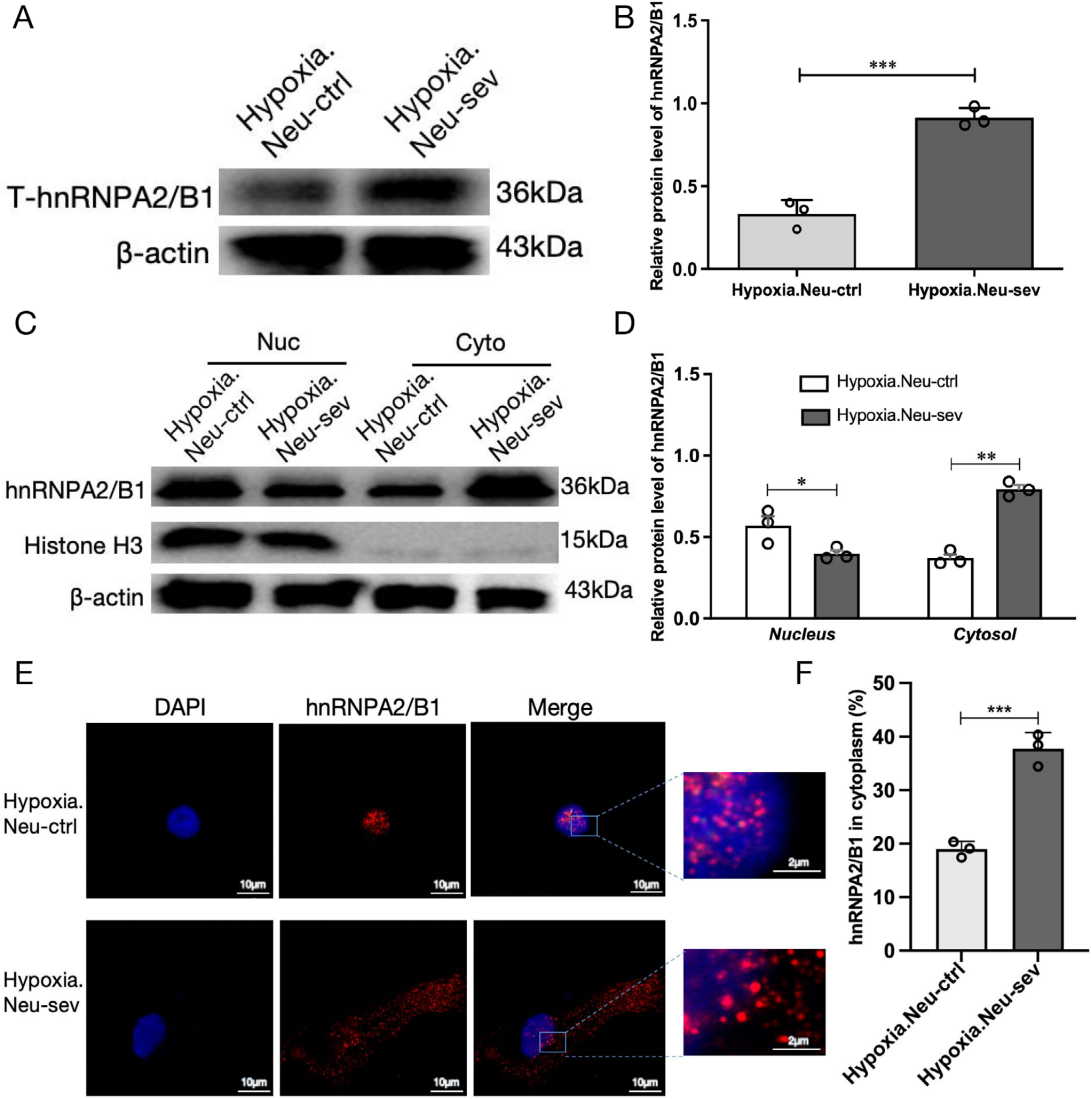
Effect of sevoflurane on hnRNPA2/B1 in hippocampal neurons under hypoxia. (A, B) Expression and statistical analysis of total hnRNPA2/B1 protein levels. (C, D) Expression and statistical analysis of hnRNPA2/B1 in the nuclei (Nuc) and cytoplasm (Cyto). (E) Immunofluorescence images of hnRNPA2/B1 (red) in the hypoxic hippocampal cells, DAPI (blue), scale bar: 10 μm，2 μm (zoom in image). (F) Quantification analysis of the cytoplasmic distribution of hnRNPA2/B1 using Image J software. Data are presented as mean ± SD (*n* = 3/group). Student's *t*‐tests was used in B, D, and F. *p < 0.05, **p < 0.01, ***p < 0.001 was considered significant.

Sevoflurane treatment significantly increased total intracellular hnRNPA2/B1 expression (Hypoxia.Neu‐sev vs. Hypoxia.Neu‐ctrl: 0.87 ± 0.24 vs. 0.37 ± 0.09; *p* < 0.001) (Figure 2A,B). Subsequently, separation of the nuclear and cytoplasmic fractions was conducted, quantitatively comparing the expression of hnRNPA2/B1 nucleoplasmic protein in sevoflurane‐treated and untreated groups. Analysis revealed a significant increase in cytoplasmic hnRNPA2/B1 levels (Hypoxia.Neu‐sev vs. Hypoxia.Neu‐ctrl: 0.75 ± 0.23 vs. 0.35 ± 0.56; *p* < 0.01) and a corresponding decrease in nuclear hnRNPA2/B1 (Hypoxia.Neu‐sev vs. Hypoxia.Neu‐ctrl: 0.40 ± 0.08 vs. 0.58 ± 0.10; *p* < 0.05) in the sevoflurane‐treated neurons compared to controls (Figure 2C,D). Mislocalized hnRNPA2/B1 accumulated at significantly higher levels in the cytoplasm of sevoflurane‐treated neurons compared to the untreated control group. This finding was confirmed by immunofluorescence confocal imaging, which revealed strong nuclear localization of hnRNPA2/B1 in control neurons, whereas neurons exposed to sevoflurane exhibited both nuclear and cytoplasmic distribution of the hnRNPA2/B1 protein (Figure 2E,F). These results suggest that sevoflurane promotes the nuclear‐to‐cytoplasmic translocation of hnRNPA2/B1 and is associated with its elevated expression.

### 3.2 Sevoflurane Triggers Hydrogel Phase Transition of hnRNPA2/B1‐SGs in Hypoxic Hippocampal Neurons

**FIGURE 3:**
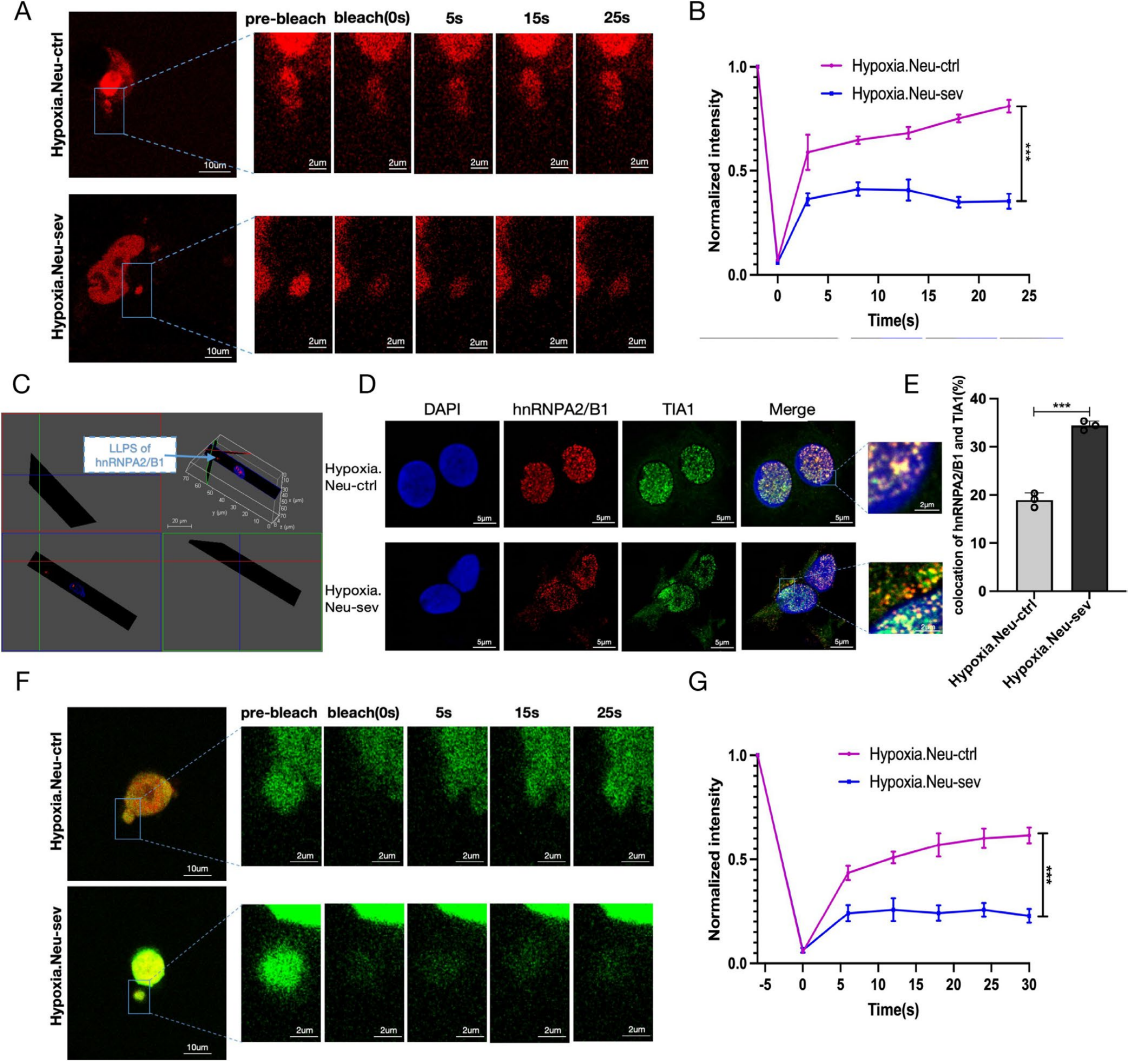
Effect of sevoflurane on the dynamics of hnRNPA2/B1 and SGs in the cytoplasm of hippocampal neurons under hypoxia. (A, B) Representative LLPS micrographs (A) and FRAP quantification (B) in the hypoxic neurons transfected with the mCherry‐hnRNPA2/B1 plasmid over a 25 s period in the absence or presence of sevoflurane. Scale bar = 10 μm low magnification; scale bar = 2 μm high magnification. (C) Spherical 3D reconstruction of hnRNPA2/B1‐mCherry condensates in the neurons. Scale bars = 20 μm. (D) Immunofluorescence images of hnRNPA2/B1 (red) and TIA‐1 (green, SGs) in the hypoxic hippocampal cells, DAPI (blue). Scale bar = 5 μm low magnification; scale bar = 2 μm high magnification. (E) Fluorescence colocalization analysis of hnRNPA2/B1 and TIA1 using Image J software. (F, G) Representative gel phase micrographs (F) and FRAP quantification (G) in the hypoxic neurons transfected with the mCherry‐hnRNPA2/B1 (red) and EGFP‐TIA1 (green) plasmid over a 30 s period in the absence or presence of sevoflurane (after colocalization of mCherry‐hnRNPA2/B with EGFP‐SG, the SGs were bleached). Scale bar = 10 μm low magnification; scale bar = 2 μm high magnification. Data are presented as mean ± SD (*n* = 3/group), and were analyzed by Student's *t*‐tests. ***p < 0.001 was considered significant.

HnRNPA2/B1 is a key nucleating protein involved in LLPS and SG formation [38]. Given its concentration‐dependent interaction with mRNA to mediate phase separation [39], we investigated whether sevoflurane affects the physiological reversible phase separation of hnRNPA2/B1 using FRAP in hypoxic neurons transfected with hnRNPA2/B1‐mCherry. Cytoplasmic hnRNPA2/B1 formed discrete puncta, and the fluorescence recovery after photobleaching was observed (Figure 3A). Three‐dimensional reconstruction further revealed spherical condensates (Figure 3C). Both results are consistent with the features of a liquid‐like phase structure and indicate LLPS. However, sevoflurane significantly reduced the fluorescence recovery rate (74% vs. 36%; *p* < 0.001) (Figure 3B), indicating impaired droplet dynamics.

Stress‐induced SG formation involves hnRNPA2/B1 recruitment [40], with the dispersed liquid phase hnRNPA2B1 concentrating as the polymeric liquid phase SGs. To further explore the properties of hnRNPA2/B1 in cells, we focused on SGs. Immunofluorescence revealed greater cytoplasmic colocalization of hnRNPA2/B1 with T‐cell intracellular antigen‐1 (TIA‐1) in sevoflurane‐treated neurons compared to controls (Figure 3D,E). Therefore, sevoflurane increased the recruitment of hnRNPA2/B1 to SGs in the cytoplasm. Co‐expression of hnRNPA2/B1‐mCherry and TIA1‐EGFP fluorescence in live neural cells and measured the mobility of droplet‐like SGs in both groups using FRAP showed markedly reduced recovery in sevoflurane‐treated neurons (59% vs. 22%; *p* < 0.001; Figure 3F,G). These results indicate that sevoflurane‐treated SGs undergo irreversible liquid‐to‐gel transition. As hnRNPA2/B1 concentration increases, cross‐β junctions form within SGs, leading to dynamic arrest and irreversible hydrogel formation, which had no fluorescence recovery after photobleaching. Thus, sevoflurane induced the aberrant hydrogel phase transition of hnRNPA2/B1‐related SGs, impairing their dynamics.

### 3.3 Kapβ2 Overexpression Restores Nuclear Import of hnRNPA2/B1 and Reverses Sevoflurane‐Induced Hydrogel Transition in Hypoxic Neurons

The nucleocytoplasmic shuttling of hnRNPA2/B1 is regulated by its C‐terminal M9 sequence PrLDs [41]. The M9 sequence functions as both a NIR and a nuclear export signal (NES), which is a smaller, strictly conserved PY‐NLS that is specifically recognized by Kapβ2 [42, 43]. Given the high affinity of Kapβ2 for the PY‐NLS and its disaggregation activity [18, 42], we investigated the effect of sevoflurane on hypoxic hippocampal neurons following Kapβ2 overexpression via adenoviral transduction.

**FIGURE 4:**
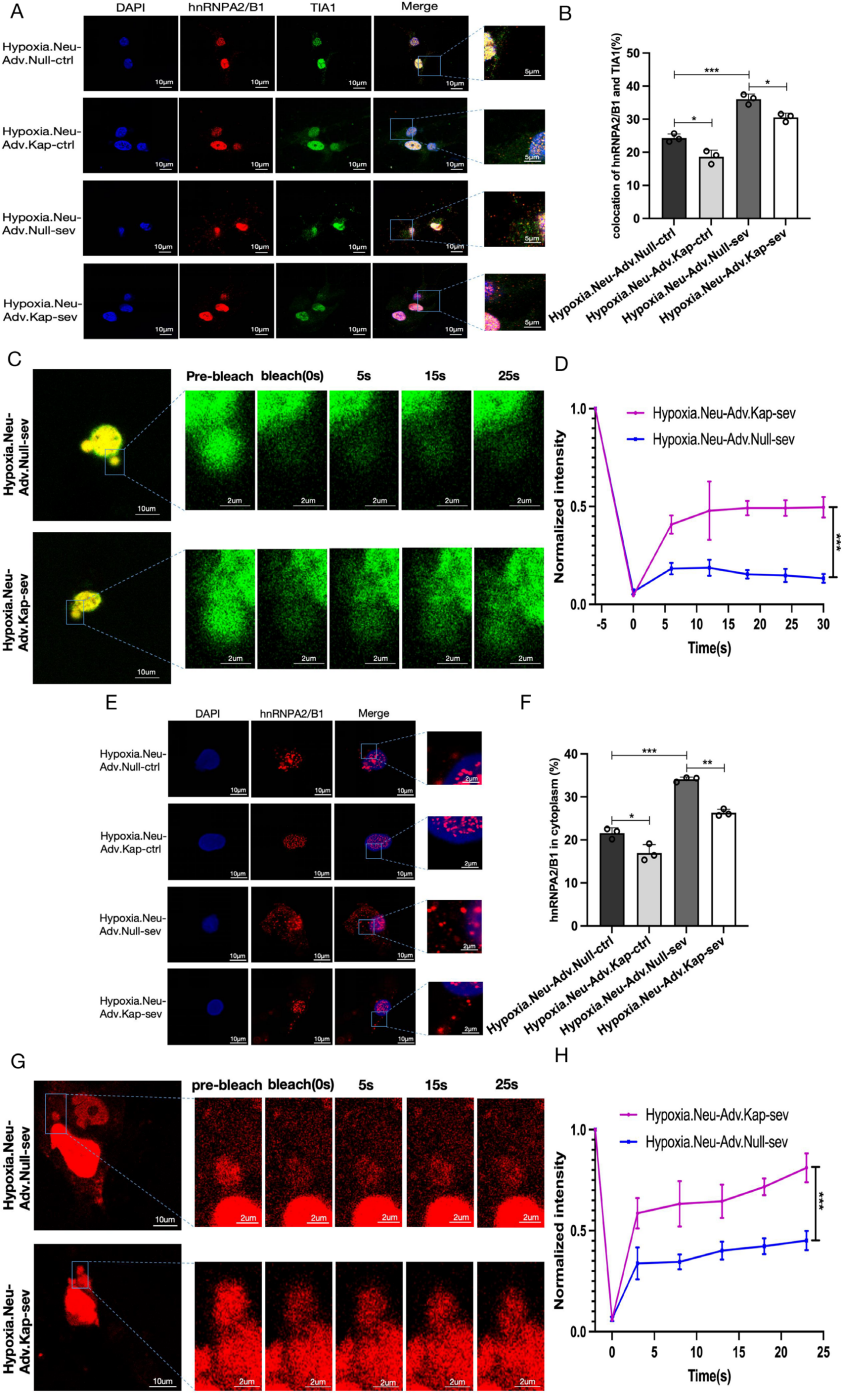
Kapβ2 mediates the nuclear import of hnRNPA2/B1 and reverses the sevoflurane‐induced hydrogel phase transition of hnRNPA2/B1‐SG. (A) Immunofluorescence images of hnRNPA2/B1 (red) and TIA‐1 (green, SGs) for hypoxic hippocampal cells infected with adv.Kapβ2 and adv. Null (MOI = 50), DAPI (blue), scale bar = 10 μm (scale bar of enlarged graph = 5 μm). (B) Fluorescence colocalization analysis of hnRNPA2/B1 and TIA1 using Image J software. (C, D) Representative gel phase micrographs (C) and FRAP quantification (D) in the hypoxic neurons infected with adv.Kapβ2 and adv. Null (MOI = 50) and transfected with the mCherry‐hnRNPA2/B1 (red) and EGFP‐TIA1 (green) plasmid over a 30 s period in different groups (after colocalization of mCherry‐hnRNPA2/B with EGFP‐SG, the SGs were bleached), scale bar = 10 μm (scale bar of enlarged graph = 2 μm). (E) Immunofluorescence images of hnRNPA2/B1 (red) for hypoxic hippocampal cells infected with adv.Kapβ2 and adv. Null (MOI = 50), DAPI (blue), scale bar = 10 μm (scale bar of enlarged graph = 2 μm). (F) Quantification analysis of the cytoplasmic distribution of hnRNPA2/B1 using Image J software. (G, H) Representative LLPS micrographs (G) and FRAP quantification (H) in the hypoxic neurons transfected with the mCherry‐hnRNPA2/B1 plasmid over a 25 s period in the absence or presence of sevoflurane. Scale bar = 10 μm (scale bar of enlarged graph = 2 μm). All data are presented as mean ± SD (*n* = 3/group). Student's *t*‐test in D and H and one‐way ANOVA in B and F were used to analyze the results. *p < 0.05, **p < 0.01, ***p < 0.001 were considered significant.

To determine whether Kapβ2 regulates the accumulation of hnRNPA2/B1 in SGs, we performed immunofluorescence staining of hnRNPA2/B1 and TIA‐1 in vitro. Overexpression of Kapβ2 markedly reduced the incorporation of hnRNPA2/B1 into SGs compared to the empty vector control, as shown by neuronal staining (Figure 4A,B). These findings indicate that upregulation of Kapβ2 reverses the recruitment of hnRNPA2/B1 into SGs. FRAP results for Kapβ2 intervention showed that neural cells co‐transfected with hnRNPA2B1‐mCherry and TIA1‐EGFP plasmid exhibited EGFP fluorescence of TIA1 recovery to some extent after photobleaching (49% vs. 14%; *p* < 0.001; Figure 4C,D), implying that Kapβ2 contributed to gel‐to‐liquid re‐transition of SGs and the liquidity and reversibility restoration of droplets.

We next assessed whether Kapβ2 promotes the nuclear localization of hnRNPA2/B1. Fluorescence imaging revealed that sevoflurane‐treated neurons overexpressing Kapβ2 exhibited increased nuclear and decreased cytoplasmic localization of hnRNPA2/B1 compared to neurons lacking Kapβ2 overexpression (Figure 4E,F). Additionally, the fluorescence recovery rate of phase‐separated hnRNPA2/B1 droplets was significantly higher in the Kapβ2‐treated group than in the empty vector group following photobleaching (71% vs. 41%; *p* < 0.001) (Figure 4G,H), indicating that Kapβ2 enhances depolymerization and nuclear import of hnRNPA2/B1, thereby improving the dynamics of phase‐separated hnRNPA2/B1 condensates in sevoflurane‐treated hypoxic neurons.

### 3.4 Kapβ2 Overexpression Enhances the Restoration of hnRNPA2/B1 and Cognitive‐Related Protein Expression in Sevoflurane‐Treated Hypoxic Neurons

**FIGURE 5:**
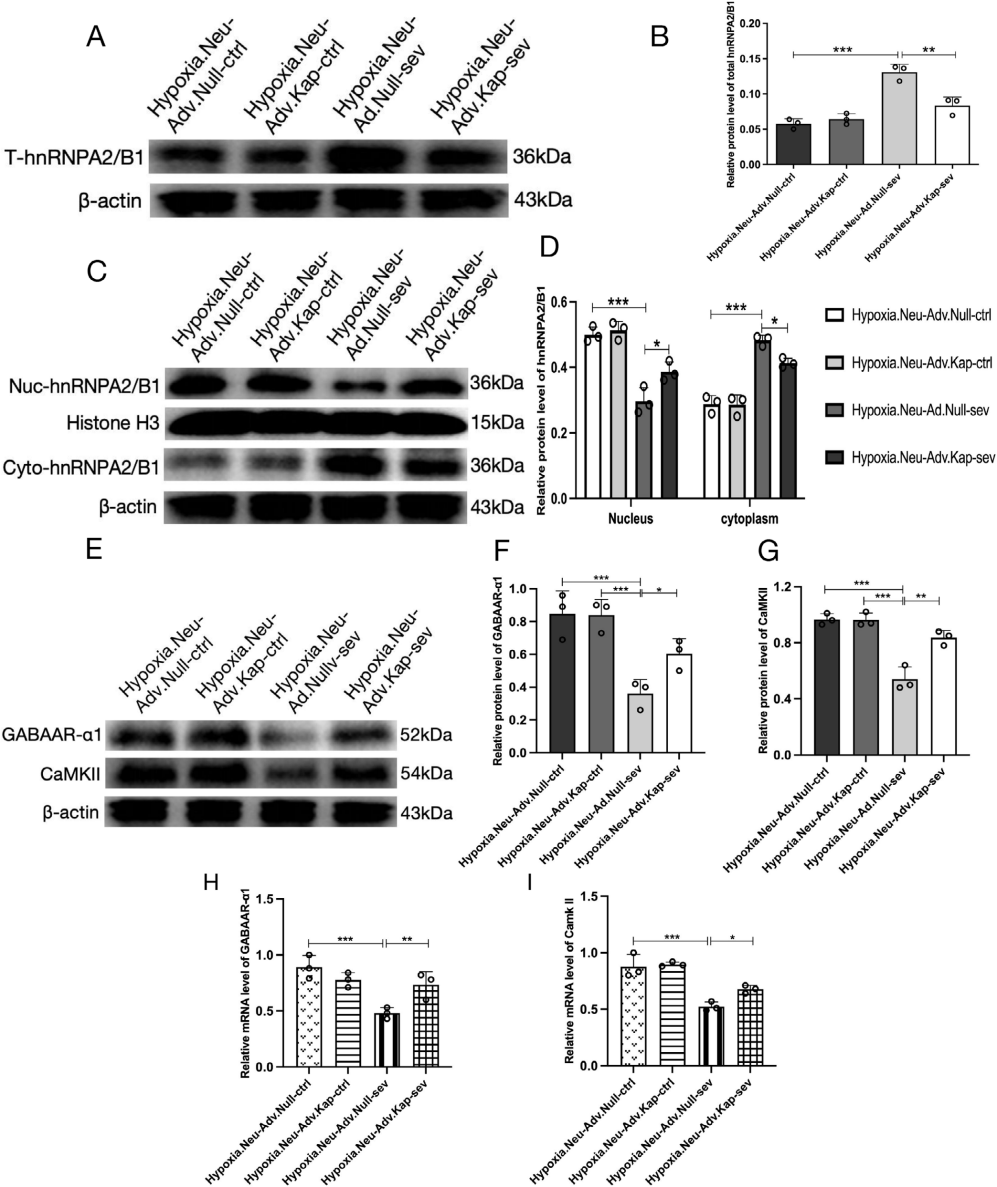
Effect of Kapβ2 on hnRNPA2/B1 and hnRNPA2/B1‐related proteins in the hippocampal hypoxic neurons (A, B) Expression and statistical analysis of total hnRNPA2/B1 protein in the hypoxic hippocampal neurons infected with Adv.Kapβ2 and Adv.Null (MOI = 50). (C, D) Expression and statistical analysis of hnRNPA2/B1 in the nuclei (Nuc) and cytoplasm (Cyto) after hypoxic hippocampal neurons infected Adv.Kapβ2 and Adv.Null (MOI = 50). (E) Expression and (F, G) statistical analysis of *GABAAR‐α1* and *CaMKII* proteins in the hypoxic hippocampal neurons infected with Adv.Kapβ2 and Adv.Null (MOI = 50); (H) qPCR analysis of *GABAAR‐α1* mRNA and (I) *CaMKII* mRNA in the hypoxic hippocampal neurons infected with Adv.Kapβ2 and Adv.Null (MOI = 50). Data are presented as mean ± SD (*n* = 3/group), one‐way ANOVA was used to analyze results of qPCR and blots. *p < 0.05, **p < 0.01, ***p < 0.001 were considered significant.

Western blot analysis revealed that Kapβ2 overexpression significantly reduced total hnRNPA2/B1 expression in sevoflurane‐treated neurons (Hypoxia.Neu‐Adv.Kap‐sev vs. Hypoxia.Neu‐Ad.Null‐sev: 0.073 ± 0.01 vs. 0.13 ± 0.02; *p* < 0.01) (Figure 5A,B). Cytoplasmic hnRNPA2/B1 was decreased (0.39 ± 0.04 vs. 0.49 ± 0.05; *p* < 0.05), while nuclear levels increased (0.38 ± 0.03 vs. 0.28 ± 0.05; *p* < 0.05) with Kapβ2 treatment (Figure 5C,D).

HnRNPA2/B1 is involved in the shuttling of pre‐mRNA transport particles from the nucleus to the cytoplasm. These transport particles carry numerous cognition‐related pre‐mRNAs, including those encoding calcium/calmodulin‐dependent protein kinase II (*CaMKII*) and the γ‐aminobutyric acid A receptor α subunit (*GABAAR‐α*) [44]. hnRNPA2/B1 regulates metabolic maturation processes of these pre‐mRNAs in the transport particles, including splicing, post‐transcriptional processing, and translation [45]. Under conditions of sustained cytotoxic stress, the post‐transcriptional processing function of hnRNPA2/B1 becomes impaired. This leads to the generation of immature, non‐functional mRNAs and the aberrant translation of faulty proteins, which accumulate in SGs. Consequently, normal protein synthesis is substantially impaired [46, 47].

To evaluate the functional recovery of hnRNPA2/B1 following Kapβ2 overexpression in vitro, we examined the expression levels of its downstream mRNA and protein targets that are carried and translationally regulated. In the Hypoxia.Neu‐Adv.Kap‐sev group, the protein levels of CaMKII and GABAAR‐α1 were upregulated compared to the Hypoxia.Neu‐Adv.Null‐sev group (CaMKII: 0.5 ± 0.06 vs. 0.81 ± 0.01, *p* < 0.01; GABAAR‐α1: 0.36 ± 0.02 vs. 0.59 ± 0.06, *p* < 0.05; Figure 5E–G). Similarly, the mRNA expression levels of hippocampal *CaMKII* and *GABAAR‐α1* were higher in the Hypoxia.Neu‐Adv.Kap‐sev group compared to the Hypoxia.Neu‐Adv.Null‐sev group (CaMKII: 0.52 ± 0.03 vs. 0.65 ± 0.04, *p* < 0.05; GABAAR‐α1: 0.48 ± 0.02 vs. 0.7 ± 0.05, *p* < 0.08; Figure 5H,I). Therefore, Kapβ2 overexpression increases *CaMKII* and *GABAAR‐α1* at the protein and mRNA levels, suggesting that the function of hnRNPA2/B1 is restored and normalized in cognitive‐related protein synthesis.

## 4 Discussion

The major finding of the current study is that sevoflurane exacerbates hippocampal neuronal dysfunction under hypoxic conditions by inducing the cytoplasmic mislocalization of hnRNPA2/B1, which drives abnormal liquid–liquid phase separation (LLPS) and subsequent irreversible hydrogel transition of hnRNPA2/B1‐containing stress granules (SGs). Crucially, we demonstrate that Kapβ2, a nuclear import receptor, reverses these pathological effects by dissolving aggregated hnRNPA2/B1, restoring its nuclear localization, and reestablishing the dynamic reversibility of SGs. In the context of the increasing number of elderly patients with chronic cerebral ischemia and hypoxia requiring surgery under sevoflurane anesthesia, our study bridges the gap between anesthesia‐related neuronal injury and the biophysical dynamics of RNA‐binding proteins, providing novel insights into the molecular interplay that may contribute to neurodegenerative disease progression. Previous studies have reported contrasting effects of sevoflurane on the brain. For instance, in the context of hemorrhagic shock, sevoflurane postconditioning was found to exert neuroprotective effects by improving mitochondrial function in the hippocampus [48]. Conversely, another study demonstrated that sevoflurane induces neurotoxicity by downregulating the protein levels of sirtuin 1 (SIRT1) and brain‐derived neurotrophic factor (BDNF) in the hippocampi of developing mice [49]. These discrepancies highlight the context‐dependent effects of sevoflurane, necessitating deeper investigation into its role in cerebral ischemia–hypoxia injury.

In this study, we investigated the cellular response of hypoxic hippocampal neurons to sevoflurane, focusing on the subcellular localization of hnRNPA2/B1 through a neuronal hypoxic model. Western blotting and immunofluorescence revealed that sevoflurane induces increased cytoplasmic translocation of hnRNPA2/B1. Moreover, hnRNPs in the cytoplasm can undergo phase separation through interactions between IDR domains, separating from aqueous solutions into protein‐rich droplet structures [15]. Consistent with our findings, increased cytoplasmic abundance of hnRNPA2/B1 was accompanied by fluorescence recovery after photobleaching of hnRNPA2/B1‐mCherry, indicating LLPS occurrence and formation of dynamic reversible droplets. Meanwhile, the fluorescence intensity recovery rate was significantly reduced in the sevoflurane‐treated group, suggesting increased droplet viscosity and reduced dynamic exchange of droplets.

Under the pathological conditions, RBPs can accumulate in the cytoplasm and form SGs [50, 51]. The physical basis of SG formation is LLPS, droplet structures of which are mainly driven by the interactions among multivalent hnRNPs containing IDRs [21]. SGs formed through LLPS can further mature into gel‐like solid states, modulated by factors such as RNA species, RBP concentrations, and crowding agents. These granules undergo time‐dependent growth, coarsening, and alterations in their dynamics and reversibility [10]. Inappropriate hydrogel transitions of SGs are associated with neurodegenerative diseases [17]. In this study, we observed a significant increase in cytoplasmic hnRNPA2/B1‐TIA1 co‐localized puncta in sevoflurane‐treated neurons compared to untreated controls under hypoxic conditions. Following fluorescence bleaching, EGFP‐TIA1 signals failed to recover, indicating that sevoflurane promotes the recruitment of hnRNPA2/B1 into SGs and induces their transition from a dynamic, liquid‐like state to an abnormal, solid‐like hydrogel state. This transition compromises the fluidity and reversibility of SGs.

**FIGURE 6:**
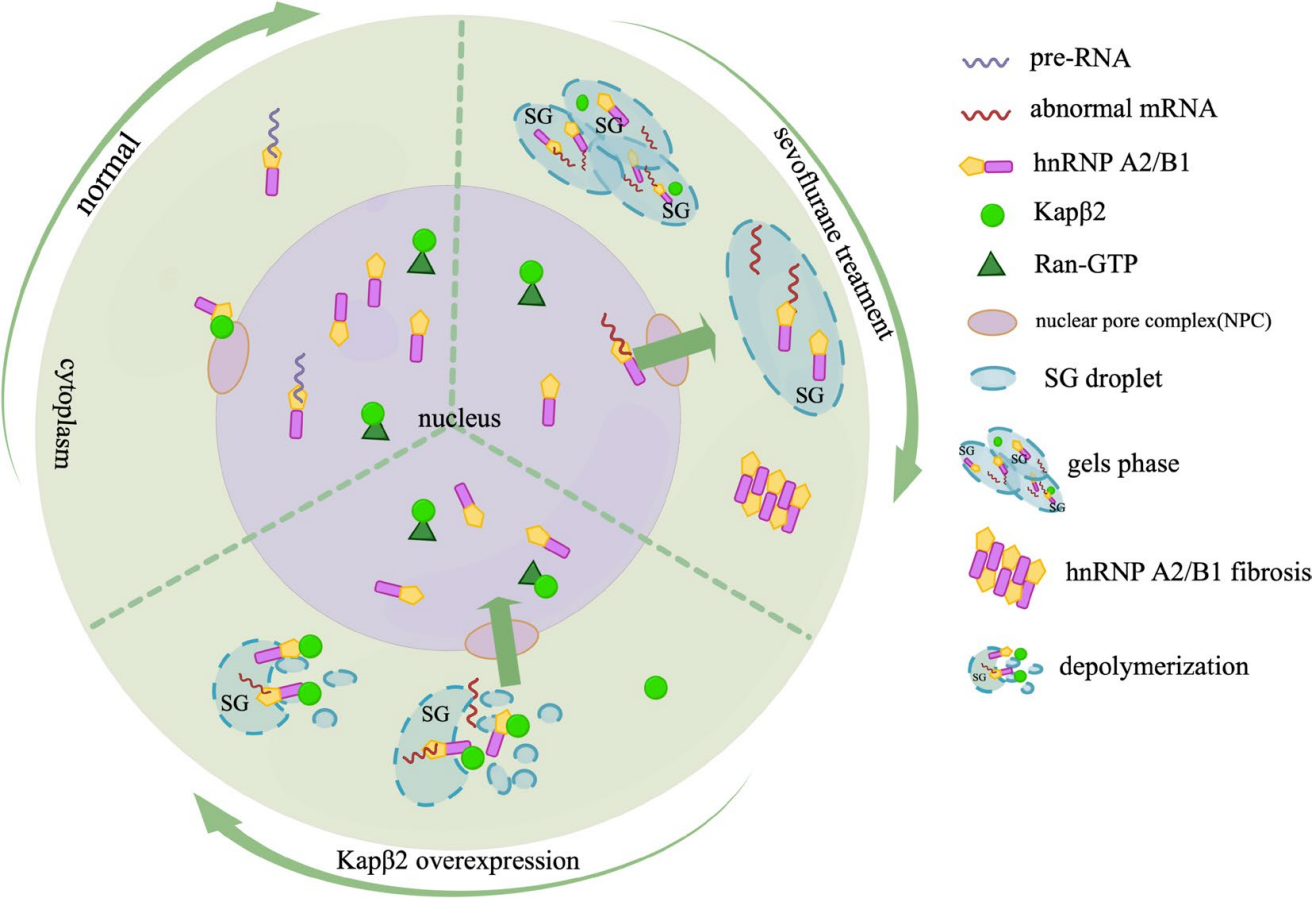
Diagram of the mechanism by which sevoflurane triggers hydrogel phase transition of hnRNPA2/B1‐SG in hypoxic hippocampal neurons, and Kapβ2 attenuates its effects.

From a therapeutic perspective, we identified Kapβ2 as a potent modulator. It, as the dominant nuclear transport factor, can recognize PY‐NLS and PrLDs of hnRNPA2/B1 [31]. In this experiment, we investigated the mechanism by which Kapβ2 reverses sevoflurane‐induced hydrogel phase transition of hnRNPA2/B1‐SG through in vitro experiments. HnRNPA2/B1 was predominantly localized in the nucleus of hypoxic neurons following Kapβ2 treatment. Co‐localization with SGs was markedly reduced, indicating that Kapβ2 reverses the recruitment of hnRNPA2/B1 by SGs and reduces the agglutination of hnRNPA2/B1 inside SGs. FRAP assays further demonstrated that the fluorescence intensity of both mCherry‐hnRNPA2/B1 and EGFP‐TIA1 was restored after fluorescence bleaching in Kapβ2‐treated neurons. These results demonstrate that in sevoflurane‐treated hypoxic neurons, Kapβ2 targets cytosolic hnRNPA2/B1, disrupting intermolecular cross‐β junctions and multivalent weak forces and dismantling agglomerated hnRNPA2/B1 in SGs. Kapβ2 promotes the reversion of SGs from an insoluble gel‐like state to a more fluid, reversible phase (Figure 6).

Western blotting demonstrated that Kapβ2 significantly decreased sevoflurane‐induced cytoplasmic hnRNPA2/B1 expression and increased nuclear expression of hnRNPA2/B1 in the sevoflurane‐induced hypoxic neurons. This finding verified Kapβ2 corrected nucleoplasmic mislocation of hnRNPA2/B1. We further evaluated the restoration of hnRNPA2/B1 function by examining the mRNAs and protein expression of *CaMKII* and *GABAAR‐α1* downstream of hnRNPA2/B1 in Kapβ2‐treated hypoxic neurons. Our findings revealed that *CaMKII* plays a prominent role in hippocampal long‐term potentiation and hippocampus‐dependent memory [52]. *GABAAR‐α1* is involved in memory formation or consolidation and connects emotional learning and memory [53]. This study found that mRNA and protein expression of *CaMKII* and *GABAAR‐α1* were significantly decreased in sevoflurane‐treated hypoxic neurons. Gene and protein expression levels of *CaMKII* and *GABAAR‐α1* increased after the upregulation of Kapβ2, indicating that the function of hnRNPA2/B1 in regulating the expression of cognitive‐related mRNAs and proteins was restored.

Although our findings achieved statistical significance with robust effect sizes (Cohen's *d* > 1.0), we acknowledge that the sample size (*n* = 3/group) in our neuronal cultures may limit the detection of subtler effects (< 20% variation). This reflects inherent biological variability in primary cultures and the technical challenges associated with quantifying weak molecular interactions. To address this limitation and validate clinical relevance, we are expanding to *n* ≥ 6 in ongoing murine studies. These experiments will assess both the reversal of hydrogel SGs and behavioral outcomes, providing a more comprehensive understanding of Kapβ2's therapeutic potential.

## 5 Conclusions

In summary, our study demonstrates that sevoflurane induces the ectopic cytoplasmic accumulation of hnRNPA2/B1, driving aberrant phase separation and irreversible hydrogel transitions of hnRNPA2/B1‐associated SGs. This impairs the transcriptional regulatory function of hnRNPA2/B1 and contributes to the dysregulation of cognition‐related proteins in hippocampal neurons. Importantly, Kapβ2 overexpression reverses these pathological effects by dissolving aggregated hnRNPA2/B1, preventing abnormal LLPS, and restoring its nuclear localization. This rescues the dynamic and reversible properties of SGs and reestablishes neuronal function. Our findings link sevoflurane‐induced neurotoxicity to the biophysical behavior of hnRNPA2/B1‐SGs and propose that hydrogel phase transition is a key pathological mechanism in sevoflurane‐induced neurodegenerative disease exacerbation. These insights lay the foundation for exploring Kapβ2 as a therapeutic strategy for mitigating sevoflurane‐induced cognitive dysfunction.

## Author Contributions

Project conception: Haiyun Wang; Study design: Haiyun Wang, Miao Zhang, Xinyi Wang; Performance of experiments: Miao Zhang, Xinyi Wang, Feiyu Jia, Lin Zhang; Initial data collection and analysis: Miao Zhang, Feiyu Jia, Chenyi Yang, Xi Xin; Final data analysis: Miao Zhang, Zixuan Wang, Huihui Liao; Writing of paper: Miao Zhang; Critical revision of paper: all authors; Supervision of experiments: Haiyun Wang, Chenyi Yang.

## Conflicts of Interest

The authors declare no conflicts of interest.

## Supporting information


Appendix S1.

## Data Availability

The data that support the findings of this study are available from the corresponding author upon reasonable request.
