# Supplementary material for: Kapβ2 Reverses Sevoflurane‐Induced Hydrogel Phase Transition of hnRNPA2/B1‐SG in Hypoxic Primary Rat Hippocampal Neurons
Source: CNS Neurosci Ther. 2025 Aug 7;31(8):e70532. doi: 10.1111/cns.70532 (PMC12331528; doi:10.1111/cns.70532)

the original, uncropped image of each blot appearing in the manuscript

Note: The lanes of the unedited gel/blot that appear in the cropped image in the manuscript are highlighted with red rectangles, and other lanes of the unedited gel/blot are highlighted with blue rectangles, representing triplicate experiments.

Full unedited blot for Figure 1A

β-actin

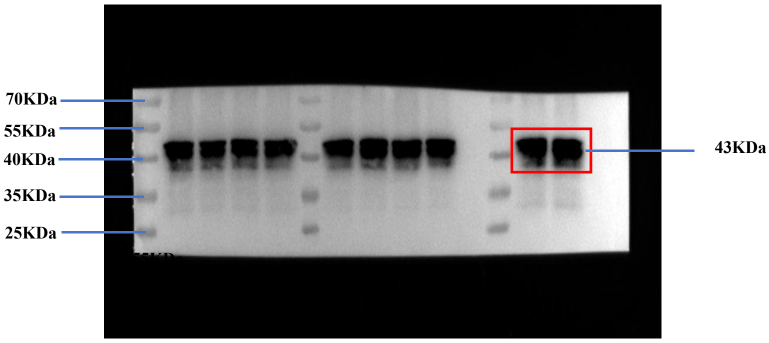

hnRNPA2B1

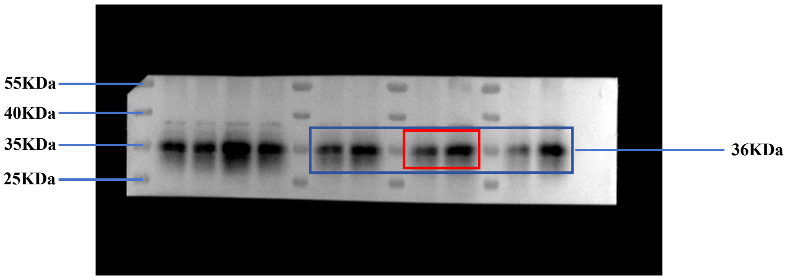

Full unedited blot for Figure 1C

β-actin

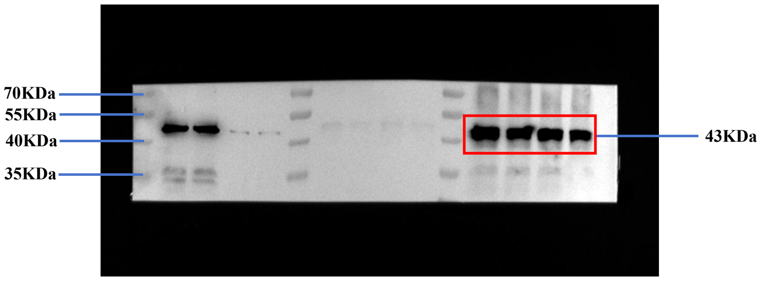

Histone H3

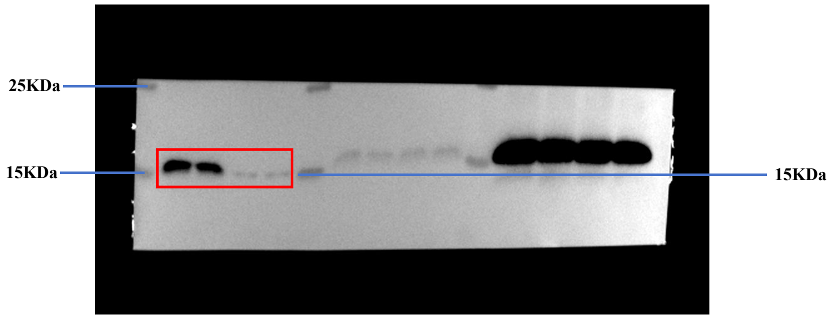

hnRNPA2B1

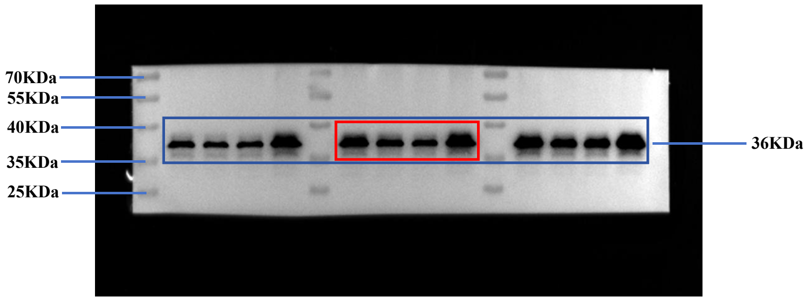

Full unedited blot for Figure 3B

β-actin

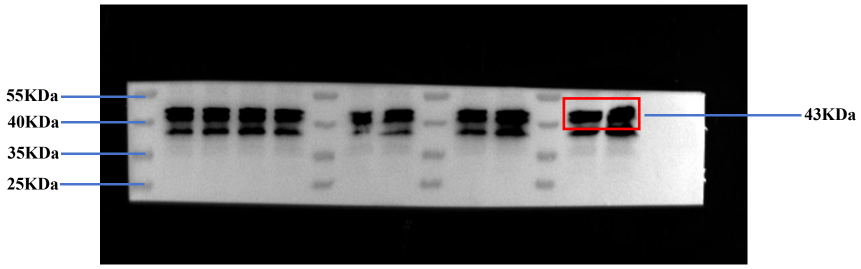

Kapβ2

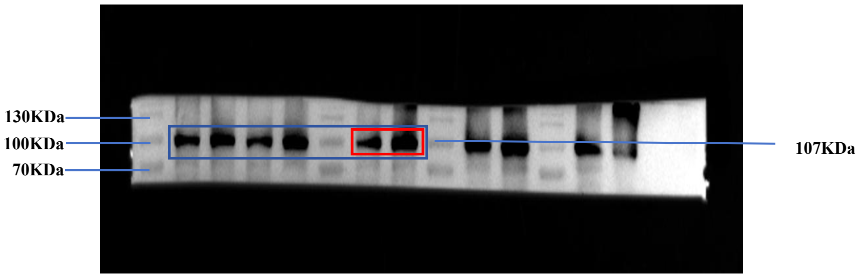

Full unedited blot for Figure 5A

β-actin

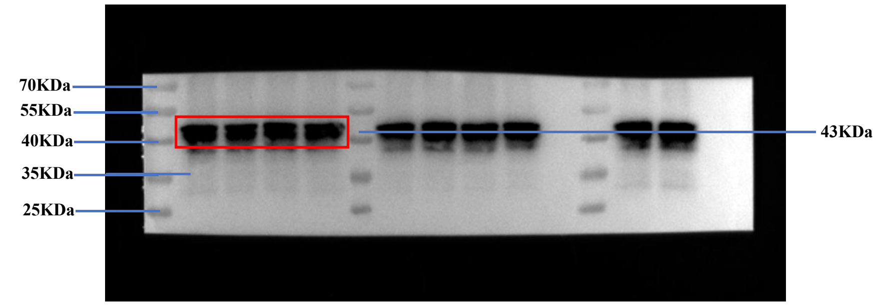

T-hnRNPA2B1

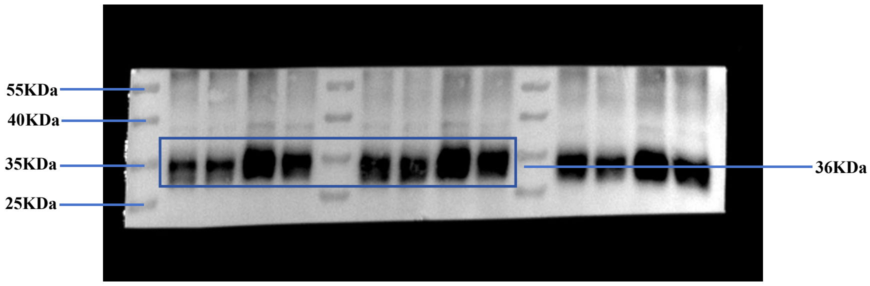

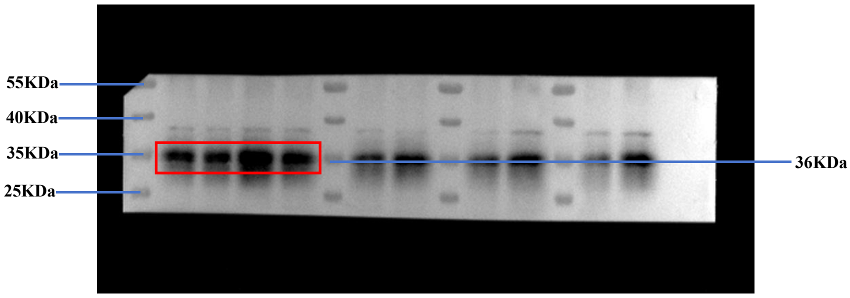

Full unedited blot for Figure 5C

β-actin

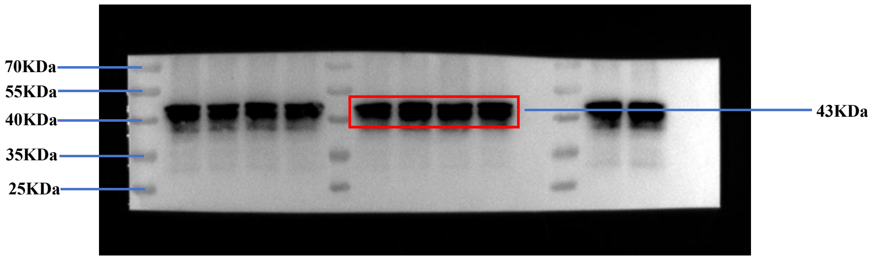

Histone H3

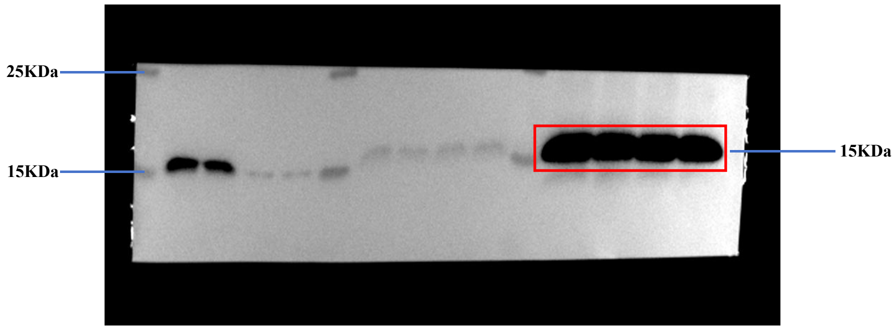

Nuc-hnRNPA2B1

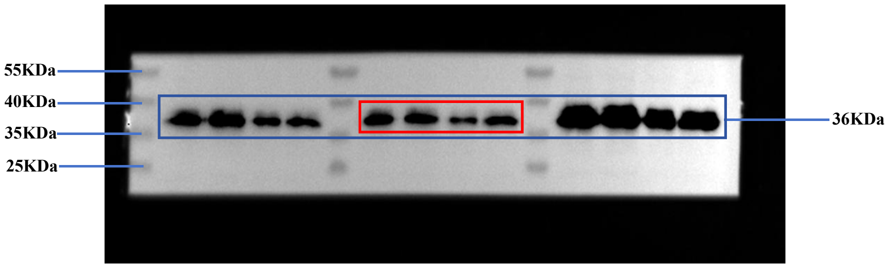

Cyto-hnRNPA2B1

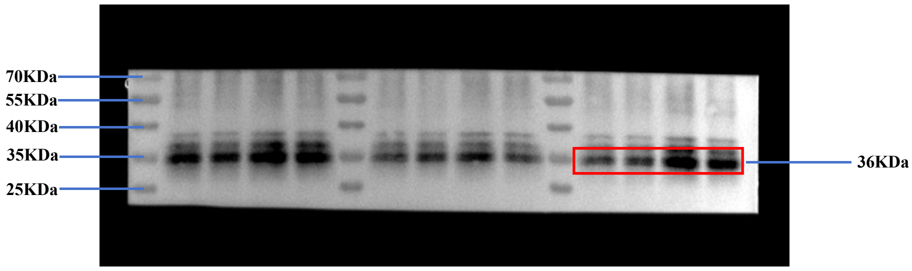

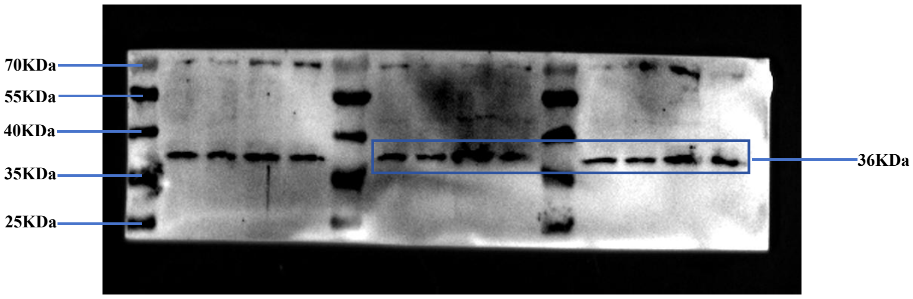

Full unedited blot for Figure 5E

β-actin

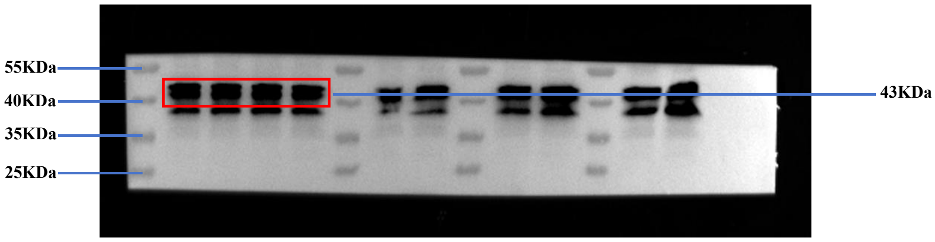

GABAAR-α1

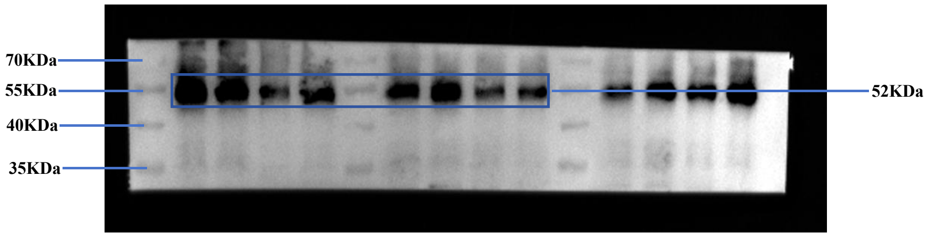

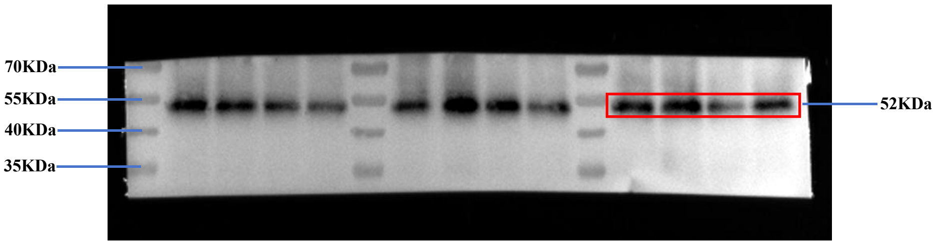

CaMKII

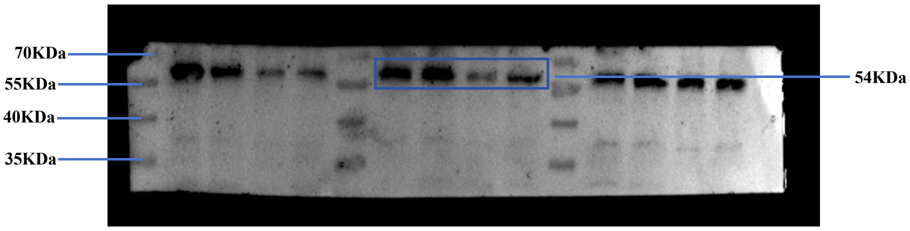

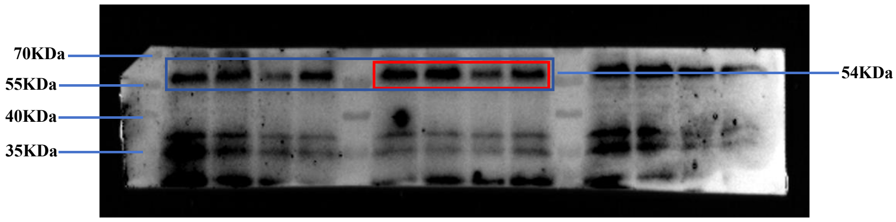

Supplement: Supplementary file 1 — Appendix S1. [file CNS-31-e70532-s001.pdf]
